# Dietary Supplementation for Fatigue Symptoms in Myalgic Encephalomyelitis/Chronic Fatigue Syndrome (ME/CFS)—A Systematic Review

**DOI:** 10.3390/nu17030475

**Published:** 2025-01-28

**Authors:** Marie Celine Dorczok, Gloria Mittmann, Nilufar Mossaheb, Beate Schrank, Lucie Bartova, Matthias Neumann, Verena Steiner-Hofbauer

**Affiliations:** 1 Research Centre Transitional Psychiatry, Karl Landsteiner University of Health Sciences, 3500 Krems, Austria; 2 Division of Social Psychiatry, Department of Psychiatry and Psychotherapy, Medical University of Vienna, 1090 Vienna, Austria; 3 Comprehensive Center for Clinical Neurosciences and Mental Health, Medical University of Vienna, 1090 Vienna, Austria; 4 Department of General Psychiatry, Vienna Health Association Clinic Ottakring, 1160 Vienna, Austria; 5 Clinical Division of General Psychiatry, Department of Psychiatry and Psychotherapy, Medical University of Vienna, 1090 Vienna, Austria; 6 Research Unit for Curriculum Development, Medical University of Vienna, 1090 Vienna, Austria

**Keywords:** myalgic encephalomyelitis/chronic fatigue syndrome, dietary supplements, PRISMA, long-COVID, chronic fatigue syndrome, clinical trials

## Abstract

**Background/Objectives**: Myalgic Encephalomyelitis/Chronic Fatigue Syndrome (ME/CFS) is a complex neuroimmunological disorder with limited treatment options. Despite the widespread use of Dietary Supplements (DSs) among ME/CFS patients to alleviate fatigue and associated symptoms, evidence remains inconclusive. This systematic review aims to provide an updated synthesis of the efficacy of DS interventions and explore possible mechanisms underlying their therapeutic effects. **Methods**: This systematic review was conducted according to PRISMA guidelines. Several databases (Ebsco Host, PubMed, Scopus, Google Scholar) were used for the systematic search, which was based on the broad search terms ME/CFS and DS with a focus on publications between 1994 and 2024. The primary outcome was fatigue, with additional considerations including psychological well-being, physical activity, and biochemical markers. Two independent researchers screened the studies for eligibility in a multi-stage process and assessed quality and bias using Cochrane’s risk of bias tools (RoB-2, ROBINS-I). **Results**: Fourteen studies (N = 809) of heterogeneous designs were included, showing a high risk of bias, mostly due to missing data and selection bias. While some interventions (L-carnitine and guanidinoacetic acid, oxaloacetate, CoQ10–selenium combination, NADH and NADH-CoQ10 combination) showed significant reductions in fatigue, methodological limitations, like small sample sizes and missing data, prevent firm conclusions. Mixed results were reported for secondary outcomes like cognitive function and inflammatory markers. Six studies noted adverse effects, including nausea and insomnia. **Conclusions**: Though some DSs showed potential in reducing fatigue in ME/CFS, methodological limitations and inconsistent results hinder definitive conclusions. Future research should improve diagnostic criteria and include more diverse populations.

## 1. Introduction

### 1.1. Myalgic Encephalomyelitis/Chronic Fatigue Syndrome

Myalgic Encephalomyelitis/Chronic Fatigue Syndrome (ME/CFS) diverges fundamentally from physiological exhaustion, representing a persistent neuro-immunological multisystem disorder characterized by non-restorative recovery [1,2]. In the context of post-COVID-19 syndrome, ME/CFS has gained heightened research interest because significant symptomatic convergence is observed [3]. Pre-COVID-19 estimates of ME/CFS prevalence in Austria ranged from 0.3–0.9%. These figures have doubled post-pandemic, with the global prevalence now estimated at 0.68% [95% CI 0.48–0.97] [4,5]. The diagnosis is disproportionately more often reported in women (3:1 female-to-male ratio) and predominantly manifests in two age cohorts (10–19 and 30–39 years). Thus, ME/CFS affects individuals in critical and active stages of their personal and professional development. [6,7,8]. Underdiagnosis has been postulated and would suggest epidemiological underestimation [9]. Research on the condition remains limited and inconsistent, reflecting its complexity and the challenges it poses.

Patients report either acute or gradual onsets, with antecedent events such as physical trauma, psychological distress, and/or infectious diseases [7]. On average, the quality of life of affected individuals is reduced to a considerable extent [10,11]. Disease severity can vary and range from mild to very severe: 75% of affected individuals are no longer able to work, and about a quarter are confined to their own homes or even beds for years [12].

Post-exertional malaise (PEM) constitutes the hallmark symptom of ME/CFS, characterized by a disproportionate and pathological response to physical or cognitive exertion [13]. Following minimal activity, patients may experience symptom exacerbation—occurring immediately or with a 12–72 h delay—potentially lasting hours to weeks, with a risk of permanent clinical deterioration [13,14,15]. PEM precipitates cascading symptomatology, including pathological fatigue, neurocognitive dysfunction (e.g., cognitive impairment, “brain fog”), sleep disturbances, emotional lability, and orthostatic intolerance [1,10].

So far, there are no useful biomarkers, and ME/CFS can only be clinically diagnosed based on a detailed medical history [14]. Collaboration between various medical specialties, such as neurology and cardiology, is essential for accurate differential diagnoses [14]. The prevalence of comorbidities in ME/CFS is remarkably high, reaching rates of up to 90%, and should be carefully considered and appropriately addressed in treatment [16]. In psychiatric evaluations, for example, it is important to distinguish ME/CFS from similarly presenting conditions like anxiety and depressive disorders [17]. Additionally, there are a number of criteria catalogs for diagnosing ME/CFS based on the symptoms present; the rather unspecific 1994 Centers for Disease Control and Prevention (CDC) Fukuda criteria (known as CDC or Fukuda criteria) [18] have been replaced by the Canadian Consensus Criteria (CCC), Criteria of the Institute of Medicine (IOM), or the International Consensus Criteria (ICC) [19,20,21,22]. Additionally, a variety of questionnaire tools (e.g., Munich Berlin Symptom Questionnaire (MBSQ) [23], DePaul Symptom Questionnaire (PSQ) [24]) are available for clinical practice.

Since 1969, the classification of ME/CFS within neurological disease frameworks has shifted, reflecting ongoing scientific ambiguity regarding its precise diagnosis and pathology. The underlying pathophysiological mechanisms of ME/CFS are still unknown [11], and effective ways to cure or even treat the disease and its associated symptoms are lacking [22]. In addition to pacing, individualized activity and energy management [25], psychotherapeutic support for secondary mental health issues [26], and recommendations for the use of off-label medications (e.g., low-dose naltrexone (LDN) [27,28]), dietary adjustments, and the use of Dietary Supplementations (DSs) are recommended for the management of ME/CFS symptoms [5,16,29].

### 1.2. ME/CFS and Dietary Supplementation

Based on Directive 2002/46/EC, dietary supplements are defined as concentrated sources of nutrients, such as vitamins and minerals, or other substances with nutritional or physiological effects intended to supplement a normal diet. Patients with ME/CFS often adopt specialized diets and add DSs to their regular food [30]. These measures might be taken to compensate for nutritional deficits caused by food intolerances or physical limitations that make it difficult to prepare or obtain a balanced diet [15]. Additionally, patients could use these strategies to manage symptoms, given the lack of evidence-based treatment options [5,14].

DSs are widely used among patients with ME/CFS despite limited and inconclusive evidence supporting their efficacy. Joustra et al. (2017) reviewed the mineral and vitamin status of ME/CFS and fibromyalgia patients, comparing them to healthy individuals and exploring potential associations between these nutritional parameters and clinical outcomes [31]. Their findings did not substantiate the hypothesis that deficiencies in vitamin and mineral status play a significant role in the pathophysiology of these conditions. However, their conclusions were limited by the substantial heterogeneity and poor quality of the included studies [31]. Campagnolo et al. (2017) reviewed a broad range of DSs, as well as changes in dietary patterns, exploring their potential role in managing ME/CFS symptoms and highlighting the lack of robust evidence for their efficacy [29]. Their findings serve as a foundational framework for this review. Therefore, in contrast to Campagnolo et al. (2017) [29], our review incorporates studies published since 2017, a period marked by increased research interest in ME/CFS, particularly due to the heightened attention following the COVID-19 pandemic [29,31,32]. In contrast to Campagnolo et al. (2017) [29], we focus only on the intake of DSs and do not focus on any additional dietary changes. This broader approach addresses both the methodological limitations and the heterogeneity of earlier studies while providing a comprehensive update on the potential role of DSs in ME/CFS management. By building on and expanding the work of Campagnolo et al. (2017) [29], this review offers an updated and nuanced perspective, addressing key gaps and advancing the understanding of DSs in the context of ME/CFS.

Notwithstanding the limitations of current evidence, some DSs have shown potential benefits for ME/CFS and related conditions. For example, nicotinamide adenine dinucleotide (NADH) and NADH-Coenzyme Q10 (CoQ10) combination supplements have been associated with symptom improvement based on the hypothesis that cellular metabolism abnormalities in ME/CFS may be alleviated through NADH intake [33,34,35]. Similarly, probiotics have been suggested to modulate gut microbiota, reduce pro-inflammatory cytokines, and enhance mucosal barrier function, offering potential benefits for ME/CFS patients [36,37]. Evidence from related chronic conditions, such as fibromyalgia, also supports the idea that regular DS use may positively influence patient health [38].

Nevertheless, the evidence supporting the efficacy of DSs in ME/CFS remains weak, owing to methodological flaws in many clinical trials. These include small sample sizes, lack of blinding, inadequate control groups, minimal effect sizes, and a lack of theoretical rationale for supplement selection. Furthermore, confounding variables, such as baseline diet and concurrent medication use, are often not controlled for. While current findings highlight areas of potential therapeutic value, they underscore the need for rigorous, well-designed studies to better evaluate the role of DSs in managing ME/CFS.

For this reason, our aim was to conduct a systematic review and provide an up-to-date overview of the efficacy of DS interventions in the management of ME/CFS symptoms. Our primary focus was on fatigue, supplemented by additional outcome measures such as psychological well-being, physical activity, and biochemical markers. Summarizing the results obtained should make it possible to deduce possible mechanisms that may explain the efficacy of DSs in ME/CFS. Systematic literature studies on ME/CFS and nutrition already exist, but these are outdated and include forms of nutrition and dietary changes, failing to focus exclusively on DSs [29,39].

## 2. Materials and Methods

### 2.1. Literature Search

The systematic review was conducted according to the Preferred Reporting Items for Systematic Reviews and Meta-Analyses (PRISMA) criteria [40]. A completed PRISMA checklist can be found in the Supplementary Materials (Table S1). The systematic search for the relevant literature was performed in the databases Ebsco Host (MedLine Ultimate, APA PsycArticles, APA PsycInfo, Psychology and Behavioral Sciences Collection), PubMed, Scopus, and Google Scholar.

Individual search strategies were applied to each database according to their specifications. The search was designed to be as broad as possible to identify all studies related to ME/CFS and DSs, including related synonyms. The following search terms were systematically used both as full text and as Medical Subject Headings (MeSH) terms: Chronic Fatigue Syndrome (which includes Myalgic Encephalomyelitis/Chronic Fatigue Syndrome, ME/CFS, CFS/ME and CFS), Diet (which includes Food, Nutrition, Nutrient, Vitamin, Mineral), and Supplement. The search results were limited to publication date (1994 to 2024), humans irrespective of age, and English language. A secondary search was also conducted by searching the included studies for further citations. An additional Google Scholar search was also performed. The search did not yield any articles that were not already found in the systematic search. The final search was conducted by two independent researchers (MCD and GM) on 7 May 2024.

### 2.2. Inclusion and Exclusion Criteria

Inclusion and exclusion criteria are shown in Table 1. Chronic fatigue occurs as a symptom or comorbidity in various diseases, including long-/post-COVID [41]. Studies involving patients with long-/post-COVID as the primary treatment group were excluded, as this review is concerned with ME/CFS as the primary disease.

### 2.3. Selection of Studies

All articles extracted from the databases were stored in Endnote 20 reference management software [42]. Full-text articles were assessed for eligibility and study quality. The process was completed in a research meeting of all involved team members, where the articles selected for inclusion in this review were discussed and confirmed.

### 2.4. Data Extraction and Quality Assessment

The included studies were read in their entirety, and relevant data were extracted. This included the following: (i) country; (ii) study design; (iii) diagnostic tool for ME/CFS; (iv) inclusion and exclusion criteria; (v) sample sizes; (vi) mean age of participants; (vii) sex and percentage of female participants; (vii) mean illness duration; (viii) intervention on treatment and control; (ix) study duration, including treatment time, washout period, and evaluation time points; (x) primary outcome measures for fatigue; (xi) primary outcomes for fatigue, including statistical significance; (xii) secondary outcome measures; (xiii) secondary outcome results, including statistical significance (if applicable); (xiv) adverse effects. Quality and bias were assessed using Risk of Bias Tools Revised Cochrane risk-of-bias tool for randomized trials (RoB-2) [43], RoB-2 for crossover trials [43], and Risk Of Bias In Non-randomized Studies of Interventions (ROBINS-I) [44]. The tools have been extensively validated, being the most frequently used quality and risk-of-bias assessment tool [45]. All results are visualized using the robvis tool [46]. The tool generates “traffic light” plots displaying domain-level assessments for each individual result, as well as bar plots showing the distribution of risk-of-bias evaluations within each bias domain.

## 3. Results

### 3.1. Overview of Studies and Study Quality

**Figure 1:**
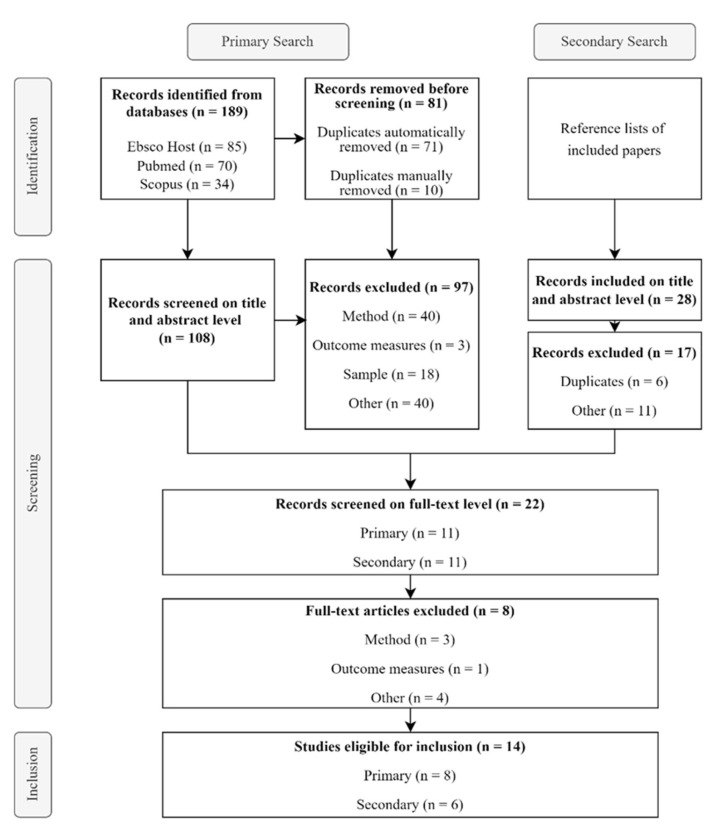
PRISMA-based flow diagram of primary and secondary search for included studies in this review of DSs on fatigue outcomes in ME/CFS.

Based on the criteria and search strategy explained above, the overall literature search identified 189 papers, of which 14 studies (7%) were included in this systematic review. Almost half of the studies (*n* = 81) were manually excluded before the first screening because they were duplicates in the different databases. A total of 43 studies were excluded because they were methodologically non-clinical studies, and 55 studies were ultimately excluded for other reasons, e.g., because they were not thematically related to either ME/CFS or DSs. Figure 1 shows the flow diagram of included and excluded studies.

Of the included studies, seven were placebo-controlled randomized controlled trials (RCTs) [33,34,47,48,49,50,51,52], of which two were cross-over designs with patients being their own controls [34,51]. One study followed a non-RCT design [53]: three groups were compared with each other and with a historical placebo group [54]. Two exploratory, open-label studies were included, one with a comparative study design without concurrent control (three-arm parallel groups) [55] and one without control group [56]. Four pilot studies were included [49,57,58,59], three of which were open-labelled [49,57,58]. However, the pilot study by Fukuda et al. [49] was a preliminary study followed by an RCT published in the same paper. The results are reported separately.

The studies differed in terms of their methodological quality and risk of bias. Almost all studies presented a high risk of bias, as shown in Figures S1–S4. Bias due to missing outcome data, as well as selection bias, was particularly common in both RCTs and NRCTs [60,61]. In NRCTs, bias due to confounding was prevalent [62].

### 3.2. Participant and Study Characteristics

The mean sample size of the treatment groups (treat) for each study was approximately 36 participants. The average age of participants (treat) was 45.6 years; children and adolescents under the age of 18 were not included in any of the studies. In total, 86% of participants (treat) were female.

The study period varied between 6 and 36 weeks, with an average time of 16 weeks. The average treatment period was 14 weeks. All participant characteristics are presented in Table 2.

The studies included evaluated a total of 14 different dietary supplements regarding their efficacy in ME/CFS. These can be categorized as follows: multi-treatments (vitamins, minerals, and coenzymes [47]; Immunovita^®^ [50]; Supradyn^®^ [56]; coenzymes, amino acids, and vitamins [58]), probiotics (Enterelle, Bifiselle, Rotanelle, Citogenex, and Ramnoselle [59]), coenzymes (CoQ10 [49]; CoQ10 and selenium [57]; CoQ10 and NADH [33,48]; ENADA^®^ [34]), amino acids (guanidinoacetic acid (GAA) [51]; acetyl-L-carnitine/propionyl-L-carnitine (ALC/PLC) [55]), alkaloids (acclydine [52]), and a supplement containing the salt oxaloacetate (anhydrous enol-oxaloacetate (AEO) [53]), which is said to be involved in the citrate cycle. Although the inclusion criteria allowed for diverse diagnostic frameworks, all studies ultimately included used the outdated 1994 Centers for Disease Control and Prevention (CDC) Fukuda criteria for the diagnosis of ME/CFS [18]. Several studies reported comorbidities among ME/CFS patients, primarily depression, anxiety, and irritable bowel syndrome (IBS). Prevalence rates varied, ranging from 24% for depression [51] to 80% for psychiatric and gastrointestinal conditions combined [33]. Other notable findings included 44% with psychiatric comorbidities [49] and 50% each for depression and IBS [58].

Relevant study characteristics are summarized in Table 3.

### 3.3. Primary Outcome: Fatigue

All included trials reported fatigue as the primary outcome. The most commonly used outcome measures were different self-report questionnaires, including the Chalder Fatigue Scale (CFQ [63]; five of fifteen trials; [49,53,58,59]) and the Fatigue Impact Scale (FIS-40 [64]; four of fifteen trials; [33,48,50,57]). In addition to the FIS-40, two trials [47,52] used a Fatigue Complaint Diary for Daily Observed Fatigue (DOF [65,66]). Other measures used were the Checklist Individual Strength-Subscale Fatigue (CIS-F [65,66,67]) [52], Patient Reported Outcome Measurement Information System Short Form Fatigue (PROMIS [68]) [53], Fibro Fatigue Scale (FFS [69]) [53,56], and Multidimensional Fatigue Inventory (MFI-20 [70]) [51,55]. Forsyth et al. [34] used a self-developed symptom scoring system based on the CDC Fukuda criteria [18].

Out of fourteen studies that evaluated different DSs for their efficacy on ME/CFS symptoms, nine showed a significant change in the primary outcome of fatigue. Five studies did not show significant changes in overall fatigue [47,49,51,52,56]. Overall, the results of the trials were very heterogeneous, making it difficult to compare them.

ImmunoVita^®^ improved cognitive fatigue significantly over 36 weeks but did not affect overall fatigue [50]. Combination supplements of vitamins, minerals, and coenzymes [47], as well as Supradyn^®^ [56], showed no significant fatigue changes. Multi-supplementation [58] and probiotics [59] both significantly reduced fatigue scores. CoQ10 and selenium [57] and CoQ10 with NADH [48] significantly reduced overall and cognitive fatigue, respectively, with ENADA^®^ showing improvement in 31% of patients [49]. GAA [51] reduced activity, motivation, and mental fatigue but not general or physical fatigue, while Carnitine [55] improved general and mental fatigue. AEO led to significant fatigue reduction for 22–33% of participants, with notable improvements for those on higher doses [53]. All primary outcome results are summarized in Table 4.

### 3.4. Secondary Outcomes

The 14 included studies assessed a wide range of secondary outcomes using equally heterogeneous instruments: health-related quality of life (SF-36 [71]), sleep quality (PSQI [72]; ISI [73]), depression (BDI I and II [74,75]; CES-D [76]; MADRS [77]), functional impairment (SIP-8 [78]), treatment response (CGI-C [79]), cognitive performance (Stroop Test [80]), pain (MPQ-DLV [81]; VAS), treatment effect (SF-12 [82]), patient’s perception of treatment effect (PGI [83]), and work and social functioning (WSAS [84]). The aforementioned tools are all self-report questionnaires. Four studies also recorded physiological parameters [47,49,51,52]. They used devices such as Life Scope [49], heart rate monitoring [49,51], and physical activity monitoring [47,51,52]. In nine of the fourteen studies, an additional laboratory analysis of the participants’ blood was performed [33,34,49,51,52,55,56,57,59]. Among other things, antioxidant status, blood count, serologic (antibody) titers, and levels of various coenzymes, vitamins, etc., were determined. Only Cash and Kaufmann [53] did not collect any variables other than the primary outcome of fatigue for the supplementation with AEO.

The results of secondary outcomes were as varied and heterogeneous as the methodologies themselves. All results for the secondary outcome measures can be found in detail in Table 5.

#### 3.4.1. Patient-Reported Outcome Measures (PROMs)

Polynutrient supplementation did not significantly impact functional impairment after ten weeks [47]. ImmunoVita^®^ improved HR-QoL and daytime dysfunction but had no effect on anxiety or depression [50]. Supradyn^®^ significantly reduced sleep disorders, autonomic symptoms, headaches, and feelings of infection, though it did not improve overall QoL [56]. Multi-supplementation showed minor improvements in sleep quality and CGI-I scale but no significant changes in HR-QoL or depression [58]. Probiotics [59] and GAA [51] significantly enhanced mental and physical health. Acetyl-L-carnitine (ALC), propionyl-L-carnitine (PLC), and their combination showed varying degrees of symptom improvement, but many patients experienced worsening symptoms during follow-up [55]. CoQ10 and selenium [57] showed an improvement in some measures of health-related quality of life (assessed by SF-36), specifically subitems emotional role functioning and mental health, whereas CoQ10 and NADH [48] increased the SF-36 subscores physical functioning, as well as sleep efficiency, quality, and duration. Bodily pain was improved by both CoQ10 combinations. Acclydine showed no significant changes compared to controls in fatigue or functional impairment [52].

#### 3.4.2. Physiological Parameters

Polynutrient supplementation did not significantly affect physical activity levels (*p* > 0.05) [47]. CoQ10 and NADH combination treatment reduced nighttime awakenings compared to placebo [33]. High frequency (HF) power, related to autonomic nervous function, decreased in the placebo group but remained unchanged in the treatment group. GAA treatment led to significant improvements in quadriceps strength and maximal oxygen uptake (*p* < 0.05) but did not affect daily energy expenditure, physical activity duration, or intensity (*p* = 0.98, *p* = 0.23, and *p* = 0.22, respectively) [51].

#### 3.4.3. Laboratory Parameters

Supradyn^®^ supplementation significantly improved superoxide dismutase (SOD) activity levels [56]. Probiotics led to increased urinary cortisol, erythrocyte sedimentation rate (ESR), and dehydroepiandrosterone (DHEA-S), with a notable rise in immunoglobulin M (IgM) levels, though some inflammatory markers like fecal calprotectin increased [59]. CoQ10 and selenium administration resulted in significant changes in low-density lipoprotein (LDL) cholesterol, Thyroid Stimulating Hormone (TSH), free thyroxine (T4), total antioxidant capacity (TAC), and reduced lipoperoxide levels, with no changes in c-reactive protein (CRP), fibroblast growth factor 21 (FGF21), or N-terminal pro-b-type natriuretic peptide (NTproBNP) [57]. CoQ10 and NADH supplementation increased NAD+/NADH, CoQ10, adenosine triphosphate (ATP), and citrate synthase levels while decreasing lipoperoxides [33]. ENADA^®^ showed no significant abnormalities in immunologic markers or oxidoreductase activity, and no significant correlation with immune function or clinical status was found [34]. GAA significantly improved muscular creatine concentrations but had no effect on other biomarkers. Acclydine showed no differences in IGF status compared to controls [52].

### 3.5. Adverse Effects and Comorbidities

Six out of fourteen studies reported adverse effects related to treatment [47,48,50,53,55,58], with two also noting side effects from placebo intake [48,50]. The most common side effects included nausea, dyspepsia, insomnia, epigastralgia, dizziness, tremors, muscle spasms, anxiety, diarrhea, and feelings of overstimulation. Interactions with other DSs or medications were not specified in any of the studies.

Certain DSs showed potential benefits for comorbidities, although these were not the primary outcomes or parameters of the studies. CoQ10 and NADH supplementation improved anxiety and depression symptoms [33,49]. Mitochondrial nutrients were associated with improved depression symptoms [58]. GAA supplementation and probiotics also improved depression and IBS symptoms, respectively [51,59].

## 4. Discussion

This systematic review compiled the existing evidence on DS interventions for fatigue and related symptoms in ME/CFS patients. The primary outcome measure for the effectiveness of DSs was fatigue, with an additional focus on quality of life, psychological well-being, physical activity levels, and various biochemical markers. Our study underscores the methodological limitations in the existing evidence and reveals a general lack of research exploring the therapeutic effects of nutritional supplementation in ME/CFS. The foundations laid by Campagnolo et al. (2017) [29] in their systematic review have been nuanced and expanded in our review.

All studies used the 1994 CDC Fukuda criteria for diagnosing ME/CFS and primarily assessed fatigue as an outcome using various scales such as CFQ [63] and FIS-40 [64]. Treatments, including supplementation with AEO [53], CoQ10 combined with selenium [57] or NADH [48], and carnitine variants [55], showed significant reductions in fatigue and improvements in other secondary outcomes, such as cognitive function and plasma L-carnitine levels. However, several studies reported no significant changes in fatigue or quality of life, highlighting the variability in treatment efficacy [47,49,51,52,56].

### 4.1. Attempts to Unravel the Complexities of ME/CFS: Exploring Immune Dysregulation, Mitochondrial Dysfunction, and Potential Therapeutic Interventions

Although the exact physical mechanisms involved in ME/CFS are not yet well understood, clinical studies have begun to provide clues. The spectrum of symptoms might be explained by immune dysregulation, microbiota dysbiosis, autoimmunity and immune priming, abnormal blood clotting and endothelial-related problems, and neurological signaling dysfunction, among other pathological mechanisms [85,86]. In the context of comorbidities, psychiatric conditions such as depression and anxiety disorders have the potential to exacerbate or overshadow symptoms [87,88].

The key symptom of ME/CFS is persistent fatigue that does not improve with rest. Mitochondria, essential for cellular energy production, generate ATP via the tricarboxylic acid (TCA) cycle and oxidative phosphorylation (OXPHOS) [89]. In ME/CFS, mitochondrial dysfunction is linked to abnormalities in structure, enzyme levels, and metabolism [90]. This results in impaired glucose and amino acid metabolism, reduced TCA cycle substrates, inefficient ATP production, and a shift toward lipid metabolism, contributing to the disease pathology [90,91]. It is important to note that the observed mitochondrial dysfunction in ME/CFS does not represent a single encircled pathology but rather a spectrum of abnormalities [92]. Persistent mitochondrial pathology is more typically linked to genetic syndromes, which are not considered a consensus feature of ME/CFS [89]. However, it is worth considering that individuals with persistent mitochondrial dysfunction could potentially have an underlying genetic issue contributing to both the mitochondrial abnormalities and the symptoms of ME/CFS [89]. While current evidence suggests that mitochondrial dysfunction in ME/CFS may arise as a secondary phenomenon, it remains unclear whether this relationship is unidirectional or if primary genetic defects could be driving the mitochondrial abnormalities [93,94]. Further research is needed to clarify whether mitochondrial dysfunction in ME/CFS is primarily a consequence or if it could also represent a contributing cause of the condition.

CoQ10, an antioxidant known for its capability to enhance ATP production, is found at reduced levels in ME/CFS patients and is linked to increased fatigue, autonomic dysfunction, and cognitive issues [95]. CoQ10 protects cell membranes and lipoproteins from oxidative damage by preventing reactive oxygen species (ROS) generation during OXPHOS and also reduces inflammation by downregulating NFκB expression [33,96]. Supplementation with ubiquinol-10, the active form of CoQ10, has been shown to alleviate fatigue and depression while enhancing cognitive function and sleep quality in ME/CFS [49]. An RCT combining NADH and CoQ10 supplementation reported improvements in fatigue, quality of life, sleep duration, maximal heart rate, ATP production, and oxidative status [97]. In their study combining CoQ10 with selenium supplementation, Castro-Marrero et al. [57] found enhanced fatigue relief, better quality of life, reduced lipid peroxidation, increased antioxidant capacity, and decreased circulating pro-inflammatory cytokines in ME/CFS patients. Even in very high doses, such supplements do not exhibit acute or chronic toxicity [98]. A natural alternative to CoQ10 obtained through fermentation could be ginseng, which has been successfully used in traditional Chinese medicine for centuries [99].

GAA supplementation increased creatine levels in muscle and serum but showed no improvement in fatigue, exercise performance, or pain, indicating creatine might not be linked to ME/CFS symptoms [51,100]. Additionally, AEO reduced fatigue in some ME/CFS patients in a small pilot trial [53]. AEO is believed to influence mitochondrial function and energy metabolism by modulating oxidative stress and reducing inflammation [101]. Metabolic studies of ME/CFS patients have already shown a deficiency of oxaloacetate in their plasma [102]. While these findings suggest AEO could be beneficial for certain ME/CFS symptoms, larger and more rigorous trials are needed to confirm its effectiveness.

A majority of ME/CFS patients experience gastrointestinal (GI) symptoms such as nausea, diarrhea, constipation, abdominal pain, and bloating. The prevalence of IBS, a functional gastrointestinal disorder, is much higher in ME/CFS compared to the general population [7,103]. Genome studies revealed altered GI microbiome structures in ME/CFS, including reduced species diversity and greater variability among patients [104,105]. Probiotics may improve GI health and reduce microbiome-related inflammation [106,107]. However, a small study on probiotics in ME/CFS showed only non-significant improvements in symptoms and inflammatory markers (i.e., IgA, IgG, IgM) [59]. However, this was a pilot study with nine participants and no control group. RCTs with more participants are needed to provide meaningful results and to make recommendations for possible treatments.

### 4.2. Addressing Bias and Improving Representation in ME/CFS Research: The Need for Larger, Inclusive Studies and Flexible Recruitment Methods

Our systematic review included fourteen studies with a total of 797 participants. Most of the participants in the studies presented were women in their mid-forties. ME/CFS can affect all age groups. Considering the mean illness duration of approximately ten years in the included studies, our results are consistent with the results of recent epidemiological studies, which state one of the two-peaked onset time points between 30–39 years of age [6,11,108]. Furthermore, diagnosing ME/CFS is inherently challenging due to the lack of diagnostic biomarkers, making it difficult to recruit patients with newly diagnosed illnesses. While studies focusing on treatment in the age group with the highest prevalence might hold more significance, the first typical onset period for ME/CFS, between 10 and 19 years of age, is entirely overlooked since no adolescents were included in any of the studies [6]. To provide evidence-based treatment recommendations, urgent research efforts that cover all age groups are needed.

Other than that, the included studies provide limited information regarding participant cohorts. With the exception of one study [59], all others at least reported age and gender distribution of participants. Additionally, a few studies mentioned participant height, weight, or BMI, as well as the duration of illness [33,34,47,48,49,53,55,58]. In clinical studies focusing on supplements and nutrition, one would expect data on participants’ dietary and activity behaviors to be collected [109,110]. Future studies should address this gap to better consider participants’ lifestyle factors and more accurately interpret “treatment” effects.

With an average of 36 participants in the treatment groups, the samples are very small. Due to a low number of participants, statistical power might get lost, and the risk of misinterpretation increases [111]. Smaller studies are also more susceptible to random outcomes [111]. Future studies should include an adequate number of participants to increase the credibility, reliability, and applicability of their research findings.

It is likely that many ME/CFS patients were excluded from the trials before they even began. Since about a quarter of affected individuals are severe to very severely impaired and house- or even bedbound, extensive physiological, psychological, and laboratory testing procedures are not feasible, and valuable participants are lost due to their intuitive exclusion from study participation [112]. So-called selection bias results in unrepresentative samples and affects the generalizability of studies’ findings [113]. This is also supported by the risk-of-bias assessments. The planning and implementation of specific studies for this group, which experiences the greatest limitations in their quality of life and health, should be considered [114]. Otherwise, future studies should at least aim to include severely affected ME/CFS patients by adopting tailored and more flexible recruitment procedures, inclusion criteria, and study protocols. Home visits, telemedical appointments, or online applications could provide support here.

### 4.3. Addressing Comorbidities in ME/CFS Research: The Importance of Including Comorbidities and Concomitant Medications for Comprehensive Treatment Evaluation

Long-COVID (also known as Post-Acute Sequelae of SARS-CoV-2 infection, PASC) is defined as a range of symptoms that continue for weeks or months after the acute phase of a COVID-19 infection has resolved [3]. These symptoms can affect multiple organ systems and significantly impair daily functioning. ME/CFS and long-COVID share many symptoms, with PEM being common to both [115]. However, some symptoms are more specific to each condition, such as decreased smell and taste, rash, and hair loss in long-COVID and painful lymph nodes, chemical sensitivities, and tinnitus in ME/CFS [116]. The diseases are closely related, and COVID and subsequent long-COVID diseases can also trigger ME/CFS [115,116].

Comorbidities are a prominent feature in ME/CFS and can significantly influence diagnosis, disease progression, and treatment outcomes [117]. Several studies identified psychiatric and gastrointestinal disorders, particularly depression, anxiety, and IBS, as the most common comorbidities, with prevalence rates ranging from frequent to very frequent (25–80%) [33,49,58]. Beyond these, other comorbidities associated with ME/CFS include fibromyalgia, Hashimoto’s thyroiditis, and myofascial pain syndrome [14,118]. These conditions can complicate clinical assessment, delay diagnosis, confound the understanding of the disease, and, in some cases, overshadow ME/CFS with other diagnoses, such as depression [117].

The interplay between ME/CFS and its comorbidities highlights the need for interventions that address the complex symptom burden of the disease. While certain DSs have shown limited promise, their effects often extend across multiple symptoms rather than representing truly targeted interventions. For instance, CoQ10 and NADH supplementation improved symptoms of anxiety and depression in ME/CFS patients [33,49], and mitochondrial nutrients were linked to improvements in depression [58]. Similarly, GAA supplementation and probiotics demonstrated beneficial effects on depression and IBS, respectively [51,59]. However, the broader effects of DSs on comorbidities remain underexplored, representing a significant gap in the literature.

Given the strong association between physical illness and mental disorders, as highlighted by Momen et al. [119], further research is essential to investigate these comorbidities in depth. This includes exploring how potential therapeutic substances or DSs that improve core ME/CFS symptoms, such as fatigue, might also alleviate other comorbid symptoms. Such research could expand the therapeutic landscape for ME/CFS and better address the multifaceted symptom burden of the disease.

However, attention must be paid to interactions [14]. For example, CoQ10 has a high interaction risk with warfarin and other anticoagulants [120], which help prevent or manage blood clots in various conditions (e.g., coronary occlusion, venous thrombosis) [121]. Information about concomitant medications, which are additional medical therapies required for the treatment of comorbidities, should be collected upon inclusion in the study and assessed and mentioned separately when evaluating study results [122]. The same applies to interactions between different DSs when used in parallel, as, for example, the interaction between Omega-3 fish oil and CoQ10 may potentially result in hypotension due to their synergistic antihypertensive effects [123].

Communication is key here. Patients must inform their physicians and study investigators of their DS intake [121]. At the same time, patients with concomitant medication or parallel use of DSs should only be excluded from study participation if clinically relevant interactions between the treatments are to be expected and the safety of participants is at risk. Rather, it is important to communicate potential interactions with patients [124].

### 4.4. Improving ME/CFS Research: The Need for Updated Diagnostic Criteria, Standardized Fatigue Assessment, and Rigorous Washout Periods in Clinical Trials

All included studies utilized the CDC Fukuda criteria for diagnosing ME/CFS [18]. However, these criteria have since been superseded by more specific diagnostic frameworks, such as ICC [19] and CCC [21]. Notably, even the more recent studies included in this review did not adopt these updated criteria. The Fukuda criteria do not require the presence of PEM for diagnosis, a limitation that has been criticized for potentially including a broad spectrum of conditions under ME/CFS [19,21]. Additionally, since the CDC Fukuda criteria require only four out of eight possible symptoms, patients can be diagnosed with minimal symptom overlap, which complicates cross-study comparability [19,125]. Future research should adopt current diagnostic guidelines to improve the classification and stratification of ME/CFS patients, allowing for better subgroup analysis. However, this transition introduces challenges in comparing the results and methodologies of studies based on the Fukuda criteria with those using the more modern frameworks [126]. Addressing this comparability issue should be a priority in future research.

A variety of instruments were used to assess changes in fatigue symptoms over the course of the studies. Apart from one study that designed its own instrument based on the CDC Fukuda criteria [34], all other studies used validated questionnaires. In their only recently published DACH consensus statement on ME/CFS, Hoffmann et al. [14] also suggest an equally broad selection of questionnaires to support the assessment of pathological fatigue in ME/CFS, e.g., Fatigue Assessment Scale (FAS [127]) and MBSQ [23]. The use of such a wide range of instruments suggests that there is no consensus on which tool is appropriate for assessing fatigue as an outcome measure in the clinical–experimental setting.

PROMs, i.e., standardized questionnaires and instruments based on subjective self-report of the participants [128], were used for both primary and secondary outcome measures. PROMs are a valuable tool but have limitations (e.g., social desirability, subjectivity, cultural and linguistic differences) that need to be considered [129]. Their results should always be interpreted in the context of other clinical data and the individual patient’s situation [130]. Careful selection and validation of the instruments used can alleviate many of these problems, as can complementary external observations and additional clinical investigations (e.g., collection of laboratory parameters) [131].

### 4.5. Variability in Washout Periods and Their Impact on Clinical Trials for DSs in ME/CFS: Urgent Need for Standardization

Clinical trials investigating DSs for ME/CFS vary widely in study design, including treatment duration, follow-up periods, and the presence or absence of washout periods. Washout periods, designed to eliminate residual effects of prior treatments, are crucial for ensuring reliable results in trials [132]. This is particularly important for ME/CFS patients, who often self-administer DSs due to the lack of effective treatment options. Without adequate washout phases, the risk of carry-over and period effects is heightened, especially in crossover studies, leading to biased outcomes. Harvey et al. [133] suggest that in addition to time-based washouts, clinical and laboratory parameters could be used to ensure patient safety and recovery from prior adverse events before enrollment.

No long-term studies on DS use in ME/CFS were included, leaving gaps in understanding the benefits and risks of prolonged use. The potential for negative effects, such as overdosing or side effects from long-term use, should not be underestimated [134,135]. For instance, selenium, a commonly used mineral supplement, has been associated with adverse effects such as vomiting, fatigue, and hair loss when taken in excess [136]. These risks highlight the need for comprehensive research into the long-term safety and efficacy of DSs, specifically in ME/CFS.

Despite the growing global DS market, which reached USD 112 billion in 2018 and is expected to double by 2028 [136], the scientific foundation for DS use in ME/CFS remains weak. Existing studies are often methodologically flawed, with high risks of bias that limit their reliability and applicability. Addressing these issues is critical to advancing the evidence base for DSs in ME/CFS, enabling supplements to contribute meaningfully to symptom management and improving the treatment landscape for patients.

### 4.6. Limitations

Fatigue is a major symptom of ME/CFS [21]. A limitation of our review is its focus on fatigue as the only primary outcome measure, which led to the exclusion of studies addressing the efficacy of DSs for broader ME/CFS symptoms, potentially omitting valuable insights into their overall therapeutic effects. Additionally, despite broad inclusion criteria allowing for various diagnostic frameworks, all included studies ultimately relied on the 1994 CDC Fukuda criteria for the diagnosis of ME/CFS [18], potentially limiting the generalizability of our findings to patients diagnosed using alternative criteria. While we reported DS dosages in the results section, we did not focus on the dosage and frequency of DS usage in the main discussion. Given the broad scope of the review, we prioritized other relevant issues, and the topic of dosages could warrant a separate article. These limitations should be considered when interpreting the findings of our review and can inspire future work.

## 5. Conclusions

This systematic review evaluated dietary supplement interventions for fatigue and related symptoms in ME/CFS patients. The primary outcome focused on fatigue, assessed through various validated questionnaires, alongside additional factors like quality of life and biochemical markers. While CoQ10 combined with NADH or selenium, NADH, L-carnitine, GAA, and oxaloacetate showed significant reductions in fatigue, inconsistencies in participant data and methodological limitations were evident in most studies. No firm conclusions can be drawn from the studies’ results due to small sample sizes and missing data. Future research should address the lack of data on participant lifestyle factors, dietary habits, and illness severity, which are crucial for understanding treatment effects, and adopt current diagnostic frameworks and standardized tools to better classify and stratify patients for meaningful insights.

## Figures and Tables

**Table 1:** Inclusion and exclusion criteria for inclusion in this systematic review based on the Population–Intervention–Comparison–Outcomes–Study (PICOS) scheme.

Inclusion	Exclusion
Studies conducted on humans, regardless of age and gender/sex; studies including participants diagnosed with ME/CFS based on current diagnostic criteria (Fukuda, International Consensus Criteria ICC, Revised Canadian Consensus Criteria CCC, Institute of Medicine Report IOM, NICE Guidelines NG206 [18,19,20,21,22])	Animal studies; studies including participants diagnosed with ME/CFS based on other criteria or patients not even diagnosed with ME/CFS
Studies assessing the efficacy of DSs (multi- or single-DS products)	Studies that used multi-treatments (e.g., DSs and cognitive behavioral therapy or pharmacotherapy)
Studies including no control group or control groups of healthy controls	Studies that explicitly compared other patient groups (e.g., depression, multiple sclerosis) with ME/CFS patients
Studies assessing the efficacy of DSs on fatigue symptoms in ME/CFS as outcome variable	Studies that used other primary outcome variables than fatigue
Intervention studies (RCTs, clinical trials, …); studies conducted and published from 1994 to the present to exclude studies conducted before the CDC Fukuda criteria [18] were published; studies available in full text; studies available in English; studies reporting original research	Non-interventional studies; studies conducted and published before 1994; studies not available in full text (even on request); studies not available in English; reviews, case reports, study protocols, duplicates

**Table 2:** Baseline characteristics of participants in treatment and control groups across included studies.

	Sample (n)		Mean Age (SD) in Years		Sex, Female %		Mean Illness Duration (SD) (years)	
Authors	Treat (n)	Cont (n)	Treat	Cont	Treat	Cont	Treat	Cont
Brouwers et al., 2002 [47]	27	26	40 (9.9)	38.9 (10.9)	74	65	8 (NR)	4.5 (NR)
Lacasa et al., 2023 [50]	29	22	52.9 (6.5)	52.5 (7.5)	100	100	NR	NR
Maric et al., 2014 [56]	36	NA	NR (18–50)	NA	36	NA	NR	NA
Menon et al., 2017 [58]	10	NA	36.3 (10.5)	NA	70	NA	11 (7.04)	NA
Venturini et al., 2019 [59]	9	NA	NR	NA	NR	NA	NR	NA
Castro-Marrero et al., 2022 [57]	35	NA	47.3 (1.5)	NA	100	NA	NR	NA
Castro-Marrero et al., 2021 [48]	72	72	45.4 (7.8)	46.8 (6.5)	100	100	15.4 (8.9)	14.7 (6.2)
Castro-Marrero et al., 2015 [33]	39	34	49.3 (7.1)	NR	100	100	15.4 (8.9)	14.7 (6.2)
Forsythe et al., 1999 [34]	26	26	39.6 (NR)	39,6	65	65	7.2 (NR)	7.2
Fukuda et al., 2016 (PIL) [49]	20	NA	36.9 (6.9)	NA	75	NA	10.3 (5.4)	NA
Fukuda et al., 2016 (RCT) [49]	17	14	34.8 (9.4)	39.5 (8.5)	77	86	NR	NR
Ostojic et al., 2016 [51]	21	21	39.3 (8.8)	39.3 (8.8)	100	100	NR	NR
Vermeulen et al., 2004 [55]	89 (ALC = 29; PLC = 30; ALCPLC = 30)	NA	ALC: 37 (11); PLC: 38 (11); ALCPLC: 42 (12)	NA	78	NA	ALC: 5.5 (NR); PLC: 3 (NR); ALCPLC: 6.0 (NR)	NA
The et al., 2007 [52]	22	22	40.9 (9.4)	43.4 (11.2)	82	82	NR	NA
Cash and Kaufmann, 2022 [53]	76 (A = 23; B = 29; C = 24)	29	47 (NR)	NR	74	NR	8.9 (NR)	NR

Abbreviations: Cont—control group/n—sample size/NA—not applicable/NR—not reported/PIL—pilot/RCT—randomized controlled trial/Treat—treatment group/SD—standard deviation.

**Table 3:** Study characteristics.

				Intervention				
Authors	Country	Study Design	ME/CFS Diagn Tool	Treat	Cont	Study Time #	Treat Time #	Washout Time #
Brouwers et al., 2002 [47]	NLD	RCT	FUK	Multi-supplement (vitamins, minerals, (co)enzymes; Numico Research BV, The Hague, The Netherlands); BID	Identical placebo, no active ingredients	12	10	NA
Lacasa et al., 2023 [50]	Spain	RCT	FUK	ImmunoVita^®^ (Vitae Health Innovation S.L., Barcelona, Spain); four capsules/d, empty stomach, 30 min. before breakfast and dinner, only with water	Identical placebo, no active ingredients	36	36	NA
Maric et al., 2014 [56]	Serbia	EXP OL SA	FUK	Supradyn^®^ (Bayer Schering Pharma, Beograd, Serbia)	None	8	8	NA
Menon et al., 2017 [58]	AUS	OL PIL	FUK	Multi-supplement (CoQ10 200 mg; ALA 150 mg; NAC 2000 mg; ALC 1000 mg; Mag 64 mg; Vit C 240 mg; Vit D3 12.5 µg; Vit E 60 IU; Vit A 900 µgREIU; vit B co-factors (B7 600 µg, B1 hydrochloride 100 mg, B2 100 mg, B3 200 mg, B5 100 mg, B6 hydrocholoride 100 mg, B9 800 mg, B12 800 mg); BioCeuticals1, Surry Hills, Australia); one tablet BID	None	16	16	NA
Venturini et al., 2019 [59]	Italy	PIL	FUK	Enterelle, Bifiselle, Rotanelle, Citogenex, Ramnoselle (all from Bromatech s.r.l., Milan, Italy); week 1: Enterelle 2 cps bid; week 2: Bifiselle 2 cps bid; week 3: Ramnoselle 2 cps bid + Enterelle 2 cps; week 4–8: Citogenex 2 cps + Rotanelle 2 cps	None	8	8	NA
Castro-Marrero et al., 2022 [57]	Spain	PIL SA OL	FUK	Bio-Quinone Active (100 mg CoQ10; Pharma Nord, Vejle, Denmark) + Seleno Precise (200 g organic selenium yeast; Pharma Nord, Vejle, Denmark); 4/d soft gel capsules CoQ10 + 1/d tablet selenium	NA	8	8	NA
Castro-Marrero et al., 2021 [48]	Spain	RCT	FUK	Combination supplement (200 mg of CoQ10 + 20 mg of NADH) + excipient (20 mg phosphatidylserine + 40 mg Vit C) four tablets/daily	Excipient (20 mg phosphatidylserine + 40 mg vit C)	12	8	4
Castro-Marrero et al., 2015 [33]	Spain	RCT	FUK	Soft gel capsules (100 mg oral CoQ10 + 10 mg NADH; Vitae Natural Nutrition S.L., Barcelona, Spain)BID	Identical placebo with no active ingredients	8	8	NA
Forsythe et al., 1999 [34]	USA	RCT CO	FUK	ENADA (10 mg NADH), two 5 mg tablets daily, orally, 1/day (45 min before breakfast on an empty stomach with a glass of water)	Equivalent placebo, two 5 mg tablet formulation	12	8 (4 cont + 4 treat)	4
Fukuda et al., 2016 [49]	Japan	OL PIL	FUK	Soft gel capsules (50 mg of CoQ10; Kaneka, Tokyo, Japan) TID (150 mg total), after meals	None	8	8	2
Fukuda et al., 2016 [49]	Japan	RA PC PA	FUK	Soft gel capsules (50 mg of CoQ10; Kaneka) TID (150 mg total), after meals	Identical placebo with no active ingredients	12	12	NA
Ostojic et al., 2016 [51]	Serbia	RCT CO	FUK	GAA (2.4 g per day), oral administration	Identical cellulose placebo, no active ingredients	32	24 (12 placebo + 12 treat)	8 (in between trials)
Vermeulen et al., 2004 [55]	NLD	EXP OL RA	FUK	2 g/d acetyl-L-carnitine OR 2 g/d propionyl-L-carnitine OR 2 g/d acetyl-L-carnitine + 2 g/d propionyl-L-carnitine	None	34	24	2 weeks
The et al., 2007 [52]	NLD	RCT	FUK	Acclydine (250 mg; Optipharma, Susteren, The Netherlands) + amino acid supplements; week 1–2, 1000 mg/d; week 3–6, 750 mg/d; week 7–8, 500 mg/d; week 9–10, 500 mg every 2 d; week 11–12, 250 mg/d; week 13–14, 250 mg every 2 d	Identical placebo, no active ingredients	14	14	NA
Cash and Kaufmann, 2022 [53]	USA	NRCT	FUK	AEO (A: 500 mg AEO BID; B: 1000 mg AEO BID; C: 1000 mg AEO TID)	Historical oral placebo	6	6	NA

Abbreviations: AEO—anhydrous enol-oxaloacetate/ALA—alpha lipoic acid/ALC—acetyl-L-carnitine/AUS—Australia/BID—twice daily (lat. bis in die)/CFS—Chronic Fatigue Syndrome/CO—cross-over/cont—control group/cps—capsular polysaccharides/CoQ10—coenzyme Q10/d—days/DO—disorder/EXP—exploratory/FUK—1994 CDC/Fukuda Criteria/GAA—guanidinoacetic acid/Mag—magnesium/NA—not applicable/NADH—nicotinamide adenine dinucleotide/NLD—Netherlands/NRCT—non-randomized controlled trial/ME/CFS—Myalgic Encephalomyelitis/Chronic Fatigue Syndrome/OL—open label/PA—parallel group/PC—placebo-controlled/PIL—pilot/RA—randomized/RCT—randomized controlled trial/SA—single-arm/TID—three times daily (lat. ter in die)/treat—treatment group/Vit—vitamin/USA—United States of America/^#^—weeks.

**Table 4:** Outcome measures and results for fatigue as the primary outcome.

**Authors**	**Outcome Measures**	**Results**
Multi-Supplements		
Brouwers et al., 2002 [47]	CIS-D FUK CODI and DOF	No significant treatment effects for self-report or behavioral measures. No significant differences between treat and cont for primary outcome measures. No change in complaints and no compete recovery at follow-up (*x*^2^ = 2, *df* (1,2), *p* = 0.36).
Lacasa et al., 2023 [50]	FIS-40	Significant improvement in the cognitive domain from baseline at the 36-week visit in the intervention group (*p* = 0.03). FIS-40 domain scores evolved in parallel between groups over the course of the study.
Maric et al., 2014 [56]	FFS	No significant change in total FFS score after treatment (*p* > 0.05). Significant decreases in fatigue (*p* < 0.0001), sleep disorders (*p* = 0.008), autonomic nervous system symptoms (*p* = 0.02), frequency and intensity of headaches (*p* < 0.0001), and subjective feeling of infection (*p* = 0.0002).
Menon et al., 2017 [58]	CFQ	Significant reduction of mean total CFQ scores (F(4,29) = 6.31, *p* < 0.001). Most notable reduction between baseline and week four (mean difference 7.66, *p* < 0.01). Nine out of eleven CFQ items improved (*p* < 0.05), with a 55% reduction in the severity of “need for more rest” (*p* < 0.01), but there were no significant improvements in “memory” and “problems starting things” (*p* > 0.05).
Probiotics		
Venturini et al., 2019 [59]	CFQ	Progressive reduction of CFQ.
Coenzymes		
Castro-Marrero et al., 2022 [57]	FIS-40	Statistically significant differences in the scores for perceived overall fatigue (*p* = 0.02). Statistically significant improvement of physical (*p* = 0.007) and cognitive fatigue perception (*p* = 0.04) at the end of intervention.
Castro-Marrero et al., 2021 [48]	FIS-40	Significant improvement of cognitive fatigue perception for treat at week four and eight from baseline (*p* = 0.005 and *p* = 0.01, respectively). Nominal improvement in the psychosocial domain for treat at week four, but no statistical significance (*p* = 0.05). Significant decrease of total FIS-40 scores at week four from baseline (*p* = 0.02), but no significant change at follow-up (*p* = 0.09 and *p* = 0.07, respectively). FIS-40 domain scores evolved in parallel between groups over the course of the study.
Castro-Marrero et al., 2015 [33]	FIS-40	Significant improvement of fatigue after eight weeks: reduction in FIS-40 total score (*p* < 0.05).
Forsythe et al., 1999 [34]	Symptom scoring system based on FUK	Present in all patients were fatigue, neurocognitive difficulties, and sleep disturbances. High-frequency symptomatology was PEM, headache, and muscle weakness; remainder had decreasing frequency of myalgias, arthralgias, and lymphadenopathy. Success rate for TREAT was 31% vs. 8% CONT (*p* = 0.05). In total, 35% of subjects were able to correctly evaluate the NADH-treatment period; 72% of study patients reported significant improvement in clinical symptomatology and energy levels at follow-up.
Fukuda et al., 2016 [49]	CFQ	PIL: no significant differences before and after treatment (*p* > 0.05). RCT: no significant differences of subjective fatigue symptoms between treat and cont (*p* > 0.05). Changes in these symptoms dependent on CoQ10 increase and OSI decrease in CFS patients after intervention.
Amino acids		
Ostojic et al., 2016 [51]	MFI-20	No effects of the intervention for general fatigue or physical fatigue (*p* > 0.05). GAA attenuated other aspects of fatigue, such as activity, motivation, and mental fatigue (*p* < 0.05).
Vermeulen et al., 2004 [55]	MFI-20	Significant improvement of general fatigue score for PLC (*p* = 0.004) and ALCPLC (*p* < 0.001). Significant improvement of mental fatigue for ALC (*p* = 0.02).
Other		
The et al., 2007 [52]	CIS-F CODI + DOF	No significant differences in change scores between treat and cont (*p* > 0.05). No significant decrease in treat for fatigue severity (CIS-fatigue +1.1 [95% CI −4.4 to +6.5, *p* = 0.7]) or functional impairment (SIP-8 +59.1 [95% CI −201.7 to +319.8, *p* = 0.65]) compared to CONT. No significant differences for fatigue severity (DOF; *p* > 0.05).
Cash and Kaufmann, 2022 [53]	CFQ FSS PROMIS-SF-7a	Reduction of measurable fatigue to score 4 or less in 22% of all patients. Drop to 4 or less on CFQ in 28% of patients in 1000 mg AEO BID treatment group and 33% of patients with 1000 mg AEO TID. Compared to historical placebo, 75% of ME/CFS participants reported an improvement in fatigue.

Abbreviations: ALC—acetyl-L-carnitine/ALCPLC—acetyl-L-carnitine and propionyl-L-carnitine/CFQ—Chalder Fatigue Scale/CFS—Chronic Fatigue Syndrome/CIS-D—Checklist Individual Strength—Subscale Depression/CIS-F—Checklist Individual Strength—Subscale Fatigue/CODI—Complaint Diary/CoQ10—coenzyme Q10/cont—control/*df*—degrees of freedom/DOF—Daily Observed Fatigue/FFS—Fibro Fatigue Scale/FIS-40—Fatigue Impact Scale/FUK—diagnostic criteria for CFS according to CDC and Fukuda et al. (1994) [18]/GAA—guanidinoacetic acid/ME/CFS—Myalgic Encephalomyelitis/Chronic Fatigue Syndrome/MFI-20—Multidimensional Fatigue Inventory/NADH—nicotinamide adenine dinucleotide/OSI—oxidative stress index/PEM—post-exertional malaise/PIL—pilot study/PLC—propionyl-L-carnitine/PROMIS-SF-7a—Patient-Reported Outcomes Measurement Information System—Fatigue Short Form 7a/RCT—randomized-controlled trial/treat—treatment group.

**Table 5:** Secondary outcome measures and results.

Authors	Secondary Outcome Measures	Secondary Outcome Results
PROMs		
Brouwers et al., 2002 [47]	FUI: SIP8	No significant differences between treat and cont for overall functional impairment (SIP8 = 182; 95% CI = −165 to 529, *p* = 0.3).
Lacasa et al., 2023 [50]	SQ: PSQI, DEP: HADS, HR-QOL: SF-36	SQ: significant improvement of daytime dysfunction for treat (*p* = 0.01, with respect to the baseline) compared to cont. Other PSQI domain scores evolved in parallel over the course of the study. DEP: no significant differences between groups over the course of the study (*p* > 0.05). HR-QOL: social role functioning improved significantly compared to the baseline for cont (*p* = 0.01). A slight reduction in cognitive fatigue symptoms was reported, along with an improvement in self-reported HR-QoL for treat. SF-36 domain scores evolved in parallel between groups over the course of the study.
Maric et al., 2014 [56]	HR-QOL: SF-36	At baseline and after treatment, no difference of HR-QOL between treat and the general population (*p* = 0.23 and *p* = 0.25, respectively). No influence of treat for HR-QOL (*p* > 0.05). CFS diagnosis alone affected diminished vitality (MD = 49.7 at both time points). Significant decreases in fatigue (*p* = 0.0009), sleep disorders (*p* = 0.008), autonomic nervous system symptoms (*p* = 0.02), frequency and intensity of headaches (*p* = 0.0001), and subjective feeling of infection (*p* = 0.0002).
The et al., 2007 [52]	FUI: SIP-8	Significant decrease in treat (SIP-8 = +59.1 [95% CI −201.7 to +319.8, *p* = 0.65]) compared to cont.
Venturini et al., 2019 [59]	HR-QOL: SF-36 DEP: BDI-I and II	HR-QOL: progressive increase of both MCS and PCS. DEP: reduction of indexes during and after probiotic protocol in comparison with the basal values.
Castro-Marrero et al., 2022 [57]	HR-QOL: SF-36 SQ: PSQI	HR-QOL: significant improvements at week eight of intervention (*p* = 0.002). Bodily pain (*p* = 0.02), emotional role functioning (*p* = 0.02), and mental health domains (*p* = 0.05) improved from baseline. SQ: no significant differences (*p* > 0.05).
Castro-Marrero et al., 2021 [48]	SQ: PSQI HR-QOL: SF-36	SQ: significant differences between treat and cont at 4-week follow-up from baseline (*p* = 0.02). Statistically significant differences for PSQI domains for treat from baseline over time (*p* < 0.05 for all). HR-QOL: physical role functioning, general health perception, vitality, social role functioning, emotional role functioning, and mental health status domains did not show any differences between treat and cont. Significant improvements in physical functioning for treat at both visits from baseline during treatment (*p* = 0.04 and *p* = 0.001, respectively). Significant improvement of bodily pain domain for treat at 4-week visit from baseline (*p* = 0.04). Reduction in vitality for cont at four-week follow-up (*p* = 0.04).
Vermeulen et al., 2004 [55]	CGI Stroop test MPQ-DLV	CGI: improvement; 59% ALC; 63% PLC; 37% ALCPLC; deterioration: 10% ALC; 3% PLC; 16% ALCPLC. Follow-Up: deterioration; 52% ALC; 50% PLC; 37% ALCPLC. No patients improved. COG: significant improvement in all groups. PAI: no significant change in any group (*p* > 0.05). Correlations of CGI improvement with MFI-20 improvement in all groups (*r* = 0.36, *p* = 0.05) and with Stroop in the ALC (*r* = 0.48, *p* = 0.01) and the ALCPLC (*r* = 0.49, *p* = 0.006), but not in the PLC group (*p* > 0.05). No correlation of CGI with PAI in any of the groups.
Ostojic et al., 2016 [51]	SF-36 PAI: VAS	Significant treatment vs. time interaction for HR-QOL (SF-36; *p* < 0.05). SF-36: significant improvement of both PCS and MCS for treat compared to cont (*p* < 0.05). PAI: no significant difference in musculoskeletal soreness over time between treat and cont (*p* > 0.05).
Fukuda et al., 2016 [49]	CES-D	PIL: significant improvements for treat, dependent on the increases in total plasma CoQ10 levels. No clinical outcomes changed over the course of the 8-week supplementation. RCT: no significant difference in depression between treat and cont (*p* > 0.05).
Menon et al., 2017 [58]	MADRS SF-12 CGI PGI WSAS SQ: ISI	DEP: no significant changes over time (F(4,32) = 1.5, *p* = 0.23). SF-12: no significant changes on the individual levels over time (*p* > 0.05). CGI: significant improvement (F(3,24), *p* = 0.01); CGI-S: no significant changes over time (F(4,33) = 1.81, *p* = 0.15). PGI: no significant changes over time (F(3,22) = 1.62, *p* = 0.33). WSAS: no significant changes over time (F(4,26) = 2.21, *p* = 0.1); SQ: significant improvement (F(4,32) = 3.55, *p* = 0.02).
Physiological parameters		
Brouwers et al., 2002 [47]	EP: AGA	No significant differences between treat and cont (*p* > 0.05).
The et al., 2007 [52]	EP: AGA	No significant differences between groups (*p* > 0.05).
Ostojic et al., 2016 [51]	EP: AGA; isometric dynamometer; treadmill; breath-by-breath metabolic system; HR monitor	Significant differences in total quadriceps isometric strength and maximal oxygen uptake between the interventions (*p* < 0.05): No differences for daily energy expenditure (*p* = 0.98), physical activity duration (*p* = 0.23), and intensity (*p* = 0.22). Trend (*p* = 0.08) towards a difference in maximal workload during ergometry between groups.
Fukuda et al., 2016 [49]	UKPT; Life Scope; HRV and beat-to-beat variation	PIL: no clinical outcomes changed over the course of the supplementation (*p* > 0.05). RCT: UKPT: Significantly improved performance for treat compared to cont (*p* < 0.05). Life Scope: significant decrease of nighttime awakenings (>1 min and 5 min) for treat compared to cont. HRV and beat-to-beat variation: significant decrease in HF power for cont but not for treat
Laboratory parameters		
Maric et al., 2014 [56]	Antioxidant status, SOD activity	SOD activity: significant correlations after treatment between SOD activity and physical aspect of HR-QOL: physical function (*r* = 0.33, *p* = 0.05), physical role (*r* = 0.37, *p* = 0.03), bodily pain (*r* = 0.43, *p* = 0.01), and total score (*r* = 0.39, *p* = 0.02). SOD and some HR-QOL mental aspects correlated after treatment: vitality (*r* = 0.37, *p* = 0.03), mental health (*r* = 0.41, *p* = 0.01), and total score (*r* = 0.43, *p* = 0.01).
Venturini et al., 2019 [59]	ESR; reactive oxygen metabolites; immunophenotyping of leukocytes; serum IgG, IgM, IgA, and IgE concentrations; UC; DHEA-S; CAL; CRP	Increase in UC (2.3×), ESR (1.7×), and DHEA-S (1.4×); reduction of about 30% of CRP values after probiotic intake. No statistical significance (*p* > 0.05). Higher basal CAL values; increased values after probiotic treatment. Significant increase of IgM (3×), but no changes in IgG and IgA serum levels. Reduction of CD4/CD8 ratio (mean index value = 1.78/2.06). d-ROM: slight reduction of mean values; great variability among patients. Patients with very low d-ROM values in T0 (Group A) increased oxidative production in T2; patients with normal d-ROM values at T0 (Group B) decreased oxidative production after treat. Group A had higher levels in BDI tests, higher CFQ, higher UC levels, and lower MCS/PCS of HR-QOL than Group B. No significant correlation between d-ROM and CFQ (*p* = 0.35, *t* =1.01), between d-ROM and BDI-I and BDI-II inventory (*p* = 0.39, *t* = 0.92; *p* = 0.18, *t* = 1.47, respectively), and between d-ROM and PCS indexes (*p* = 0.71, *t* = 0.39). Significant correlation between dROM and MCS (*p* = 0.04; *t* = 2.47).
Castro-Marrero et al., 2022 [57]	Biomarker assays (e.g., cholesterol, free T4)	Significant differences for LDL, cholesterol, TSH, and free T4. Significant increase in TAC (*p* < 0.001). Significant decrease in lipoperoxide content (*p* < 0.001) after treatment. Significant decreases in inflammatory cytokine levels (*p* < 0.01 for all) at eight-week follow-up. No significant differences in BLD CRP levels, FGF21, and NTproBNP (*p* > 0.05 for all).
Forsythe et al., 1999 [34]	RBC and WBC; erythrocyte sedimentation rate; serum chemistry; urinalysis; serum IgG, IgM, IgA, and IgE concentrations; enumeration and quantitation of CD3, CD4, CD8, CD19, and CD16/56); E1 enzyme assay; EBV-VCA; thyroid function levels; serum antibody titers to HHV-6, HIV, RF, and Hep B/C (HBS Ag, anti-HCV)	No correlation between immune function or clinical status nor treatment response. No differences in E1 activity before or after NADH treatments. 60% had EBV-EA = 40. 40% had HHV-6 titers = 1:160. 4% were anti-HCV positive. 100% were HIV-negative, and RF was negative. 13% had elevated levels of IgE. T4 and TSH levels were within normal limits for all subjects (100%). Immunologic testing was discontinued due to non-detectable abnormalities in serum immunoglobulin concentrations or lymphocyte subset analysis.
Vermeulen et al., 2004 [55]	BLD for free carnitine and carnitine esters in plasmas	Significant increase in plasma L-carnitine in all groups. Levels of the carnitine esters increased in all groups but remained low compared with L-carnitine. No sex differences. Change in the plasma L-carnitine concentration in the ALC group was inversely related to clinical improvement, but not in the other groups. Change in plasma carnitine was related to improvement of MFI-20 in the ALC group, but not in the PLC and ALCPLC group. Change in plasma carnitine was not related to change in COG or PAI. Plasma ALC and PLC were not related to CGI.
Ostojic et al., 2016 [51]	BLD and 24 h urine: serum and urinary GAA; creatine and creatinine; total serum homocysteine; RBC; WBC; platelets; hemoglobin; hematocrit; RBC indices; ESR; Glc; total cholesterol; triglycerides; lipoprotein levels; serum sodium; potassium; Ca; enzyme serum activities (AST; ALT; LDH; ALP; CK); urine protein, blood, and Glc	Significant effect of treat for all guanidino compounds (*p* < 0.05) except for urinary creatine (*p* > 0.05). After three months of treat, significant improvement of muscular creatine concentrations compared to cont (36% vs. 2%; *p* < 0.01). No effect of treat on blood Glc and lipid profiles, liver and muscle enzymes, hematological indices, and urinary analyses outcomes.
Fukuda et al., 2016 [49]	CoQ10 levels + serum oxidation activity (reactive oxygen metabolite-derived compounds) and antioxidant activity	PIL: significant increase in plasma CoQ10 levels compared to baseline (*p* < 0.05). RCT: significantly increased plasma CoQ10 concentrations (4×) for TREAT compared to CONT (*p* < 0.05). Significantly lower plasma ubiquinone levels in patients without any lifetime psychiatric disorders ([N = 13] = 0.07 ± 0.06 vs. [N = 6] = 0.17 ± 0.09; Z = −2.2, *p* = 0.03).
Castro-Marrero et al., 2015 [33]	BLD: NAD+/NADH levels + ratio; CoQ10 levels; TBARS levels; intracellular ATP; citrate synthase assay	NAD+/NADH (*p* < 0.001), CoQ10 (*p* < 0.05), ATP (*p* < 0.05), and citrate synthase (*p* < 0.05) were significantly higher. Lipoperoxides (*p* < 0.05) were significantly lower in blood mononuclear cells of treat.
No secondary measures		
Cash and Kaufmann, 2022 [53]	NA	NA

Abbreviations: AGA—actigraphic assessment/ALC—acetyl-L-carnitine/ALCPLC—acetyl-L-carnitine and propionyl-L-carnitine/ALP—alkaline phosphatase/ALT—alanine transaminase/AST—aspartate trans-aminase/ATP—adenosine triphosphate/BDI—Beck Depression Inventory I and II/BLD—blood/Ca—calcium/CAL—calprotectin/CD3/4/8/16/19/56—cluster of differentiation 3/4/8/16/19/56/CES-D—Center for Epidemiologic Studies Depression Scale/CFQ—Calder Fatigue Scale/CGI—clinical global impression of change/CGI-S—clinical global impression of change, subscale severity of illness/CI—confidence interval/CK—creatine kinase/cont—control group/COG—cognitive performance/CoQ10—coenzyme Q10/CRP—C-reactive protein/DEP—depression/DHEA-S—dehydroepiandrosterone sulfate/d-ROM—reactive oxygen metabolites test/EA—early antigen/EBV—Eppstein Barr Virus/EP—exercise performance/ESR—erythrocyte sedimentation rate/E1—oxidoreductase/FGF21—fibroblast growth factor/FUI—functional impairment/GAA—guanidinoacetic acid/Glc—glucose/HADS—Hospital Anxiety and Depression Scale/HBS Ag—hepatitis B virus surface antigen/HCV—hepatitis C virus/HF—high frequency/HHV-6—human herpesvirus 6/HIV—human immunodeficiency virus/HR—heart rate/HRV—heart rate variability/HR-QOL—health-related quality of life/IgA/IgE/IgG/IgM—immunoglobulin A/E/G/M/ISI—Insomnia Severity Index/LDH—lactate dehydrogenase/LDL—low-density lipoprotein/MADRS—Montgomery–Asberg Depression Rating Scale/MCS—mental component indexes/MFI-20—Multidimensional Fatigue Inventory/MPQ-DLV—McGill Pain Questionnaire-Dutch Language Version/NA—not applicable/NAD+ resp. NADH—nicotinamide adenine dinucleotide/NTproBNP—B-type natriuretic peptide/PAI—pain/PCS—physical component indexes/PGI—Patient Global Impressions/PLC—propionyl-L-carnitine/PROMs—patient-reported outcome measures/PSQI—Pittsburgh Sleep Quality Index/RBC—red blood cell count/RF—rheumatoid factor/SF-12/36—Short Form Health Survey/SIP-8—Sickness Impact Scale/SOD—superoxide dismutase/SQ—sleep quality/TAC—total antioxidant capacity/TBARS—thiobarbituric acid reactive substances/treat—treatment group/TSH—thyroid stimulating hormone levels/T4—thyroxine/UC—urinary free cortisol/UKPT—Uchida-Kraepelin Psychodiagnostic Test/VAS—visual analogue scale/VCA—virus capsid antigens/WBC—white blood cell count/WSAS—Work and Social Adjustment Scale.

## Data Availability

The data presented in this study are available in the original articles. These data were derived from the following resources available in the public domain: Pubmed, Scopus, and Ebsco Host. Access restrictions may apply. Datasets and original articles may be requested from the respective authors.

## Supplementary Materials

The following supporting information can be downloaded at https://www.mdpi.com/article/10.3390/nu17030475/s1, Supplementary Material S1 presents visual representations for assessing risk of bias (ROB) using risk of bias tools. Figure S1: ROBVIS ROB-2 Traffic Light; Figure S2. ROBVIS ROB-2 Bar Plot; Figure S3. ROBVIS ROBINS-I Traffic Light; Figure S4. ROBVIS ROBINS-I Bar Plot; Supplementary Material S2: The PRISMA 2020 checklist is a guide to help authors of systematic reviews and meta-analyses report their work transparently and comprehensively [40]. It consists of 27 items. Table S1: PRISMA 2020 Checklist.
