# Supplementary material for: Dietary Supplementation for Fatigue Symptoms in Myalgic Encephalomyelitis/Chronic Fatigue Syndrome (ME/CFS)—A Systematic Review
Source: Nutrients. 2025 Jan 28;17(3):475. doi: 10.3390/nu17030475 (PMC11819863; doi:10.3390/nu17030475)
Supplement: Supplementary file 1 [file nutrients-17-00475-s001.zip › Supplementary Material S1.pdf]

Supplementary Materials Figures

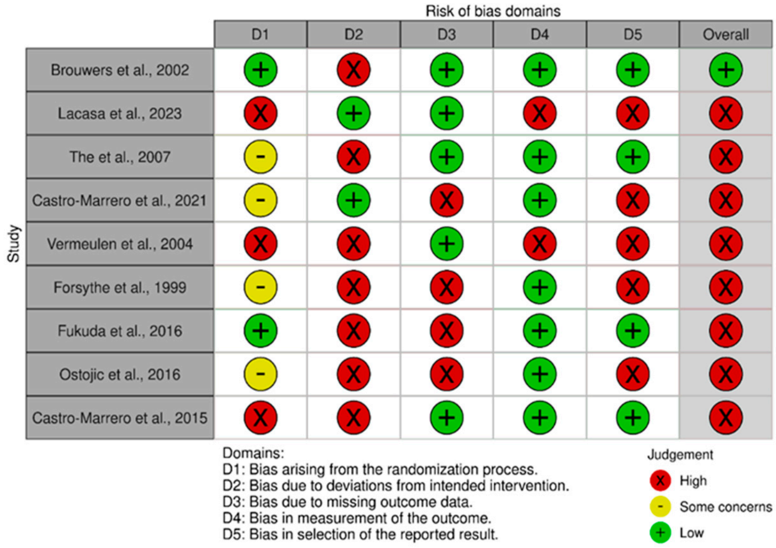

Figure S1. ROBVIS ROB-2 Traffic Light.

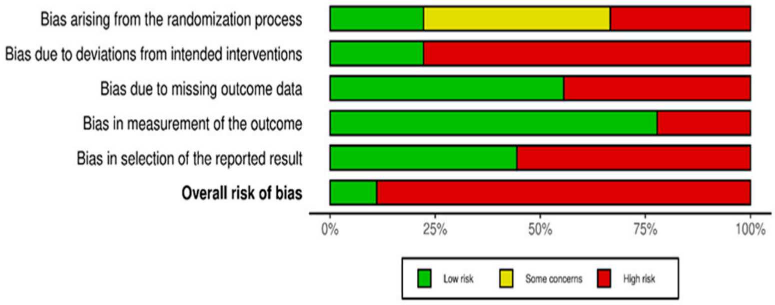

Figure S2. ROBVIS ROB-2 Bar Plot.

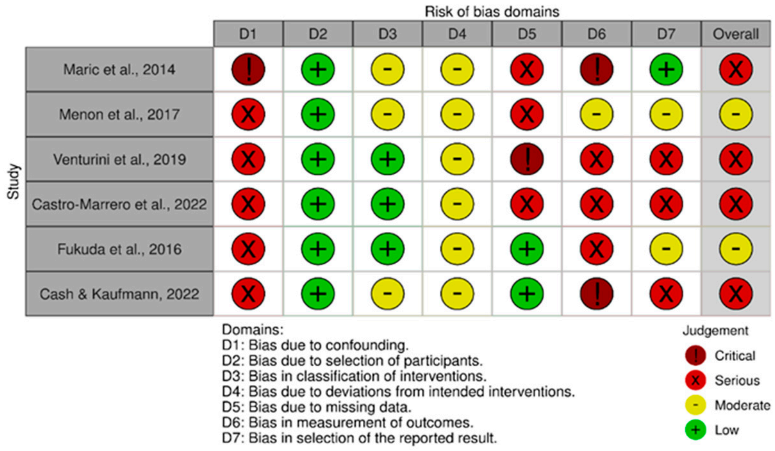

Figure S3. ROBVIS ROBINS-I Traffic Light.

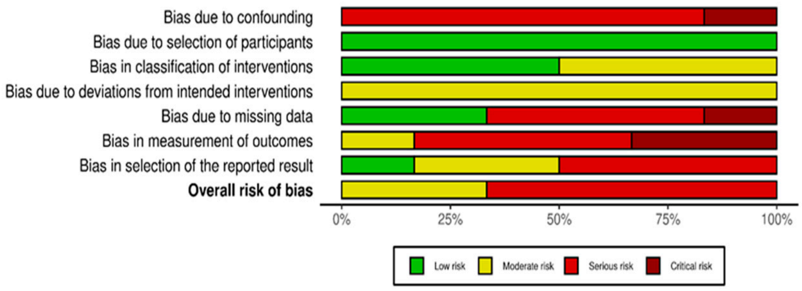

Figure S4. ROBVIS ROBINS-I Bar Plot.
